# Cassava cell wall characterization and degradation by a multicomponent NSP-targeting enzyme (NSPase)

**DOI:** 10.1038/s41598-019-46341-2

**Published:** 2019-07-12

**Authors:** Larissa Staack, Eduardo Antonio Della Pia, Bodil Jørgensen, Dan Pettersson, Ninfa Rangel Pedersen

**Affiliations:** 1 Novozymes A/S, Krogshoejvej 36, 2880 Bagsværd, Denmark; 2 University of Copenhagen, Department of Plant and Environmental Sciences, Thorvaldsensvej 40, 1871 Frederiksberg, Denmark

**Keywords:** Cell wall, Multienzyme complexes

## Abstract

Cassava (*Manihot esculenta* Crantz) is considered the third most important source of calories in tropical regions. Up to one third of cassava harvested worldwide is used in livestock production. The focus of this study was to characterize cassava cell wall structure to provide knowledge for a better application of cassava as an energy source in monogastric animal feed. A total of five cassava samples from different feed mills in South East Asia were investigated. On a dry matter basis, the cassava cell walls contained, on average, 640 mg g^−1^ glucose, 140 mg g^−1^ galactose, 50 mg g^−1^ mannose, 80 mg g^−1^ xylose, 60 mg g^−1^ arabinose, 10 mg g^−1^ fucose and 20 mg g^−1^ rhamnose. RONOZYME VP (DSM Nutritional Products, Switzerland), a non-specific multicomponent non-starch polysaccharide (NSP) degrading enzyme (NSPase) product from *Aspergillus aculeatus*, solubilized about 10% of cassava NSP content during 4 h incubations at 40 °C and pH 5. There was notable solubilization of polymers containing uronic acids, galactose, arabinose and rhamnose. Immuno-microscopy imaging indicated the solubilization of pectin, galactan and xyloglucan polysaccharides from cassava cell wall. As a consequence, the starch granules became more available to exogenous α-amylase degradation.

## Introduction

The current world population of 7.6 billion is expected to reach 8.6 billion by 2030, with Africa and Asia being the continents with the highest projected population growth^1^. Consequently, the increased demand for grains and cereals will lead to a price surge. Africa and Asia are already classified as net food importers^2^, and optimizing the application of alternative local crops to substitute costly imported raw materials is a way to soften the impact of the upcoming changes.

Cassava (*Manihot esculenta* Crantz) is the major member of the Euphorbiaceae family being cultivated as a food crop in tropical areas, with an around-the-year availability^3^. It can grow on impoverished soils and tolerate higher temperatures and drought periods^3,4^. Furthermore, cassava is the crop expected to be the least injured by the harsher climate conditions predicted for 2030, when compared to maize, millets, sorghum, banana, and beans^5^.

From 1976 to 2016, cassava global production grew from 114 to 277 million tons per year^2^. Between 11.5 and 33% of harvested cassava root is used for livestock production^6–9^. However, some concerns are yet to be overcome to efficiently introduce cassava in the feed industry. In order to optimize the utilization of cassava as energy source in livestock production, it is necessary to diminish two major disadvantages of this raw material (i) the concentration of cyanogenic glycosides that is present in all parts of the cassava plant^10^, (ii) and the effect of cassava cell walls on limiting nutrient availability, such as starch^11^. The first problem can be overcome by sun-drying chopped cassava during a couple of days, until the moisture is reduced to 100–140 mg g^−1^, which increases the product shelf-life and releases the volatile hydrogen cyanide^3^. In this project, the latter shortcoming of intact cell walls was addressed with use of an exogenous non-starch polysaccharide degrading enzyme product (NSPase).

The application of enzymes in animal feed enables the use of a wider range of raw materials, including those poorly digestible; as well as reduces variability in the nutritive value between batches of similar ingredients, thereby minimizing the variation between quality of the same raw material. Enzymes can also help to decrease viscosity of feed raw materials having high levels of NSP^12,13^; and they contribute to production of prebiotic oligomers via their action on cell wall polysaccharides, which have a positive effect on the animal gut health^12,14^. Reduction of cassava viscosity has been previously related to the degradation of homogalacturonan and released 1,4-β-D-galactan and 1,5-α-L-arabinan^15^.

The objective of the current project was to characterize the cell wall polysaccharides from different sources of cassava, and study the solubilization of the NSP content through treatment with a commercial cell wall degrading enzyme product (RONOZYME VP, DSM Nutritional Products, Switzerland), resulting in an increased exposure of starch to α-amylase.

## Results

### Compositional analysis

After grinding (0.5 mm), the compositional analysis of five cassava samples from different feed mills in South East Asia was studied (Eurofins Scientific, Belgium, Table 1). The starch content varied between 687 mg g^−1^ and 801 mg g^−1^, crude protein from 22 mg g^−1^ to 36 mg g^−1^ and crude fibre from 30 mg g^−1^ to 57 mg g^−1^ of total dry matter content. Crude fat was low in all samples. The crude ash varied between 22 mg g^−1^ and 39 mg g^−1^ for cassava samples A-D, while cassava E displayed a considerably higher content of ash (130 mg g^−1^).

**Table 1:** Compositional analysis of cassava samples.

Sample	Country of origin	Chemical composition (mg g^−1^ of dry matter)*				
		Starch	Ash	Crude fat	Crude protein	Crude fibre
A	Vietnam	801	22	4	36	36
B	Philippines	777	38	6	23	36
C	Philippines	798	39	4	22	30
D	Philippines	767	22	5	31	41
E	Philippines	687	130	6	25	57

^*^Samples analysed by Eurofins Scientific (Belgium).

### NSP analyses – cassava cell wall monosaccharide composition and solubilization by NSPase

**Figure 1:**
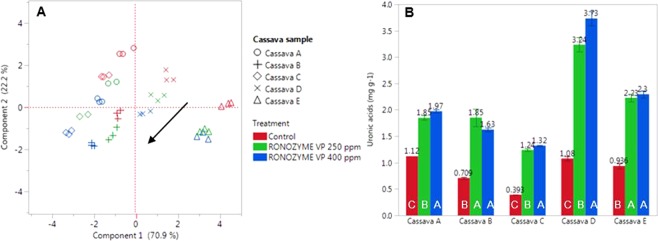
Cassava cell wall monosaccharide composition and solubilization by enzyme. **(A)** PCA plot of neutral sugars in the insoluble NSP fraction of cassava samples (A–E) before and after treatment with NSPase at 250 or 400 ppm. (**B)** Soluble uronic acids quantified by HPIC in cassava samples (A–E) before (red bars) and after treatment with NSPase at 250 (green bars) or 400 ppm (blue bars). Different capital letters within each cassava sample in the graph indicate statistical differences between enzyme treatments (Tukey-Kramer P < 0.05).

The insoluble neutral sugar composition (on a dry matter basis) of the cassava cell wall polysaccharides is shown in Table 2. On average, cassava total NSP is constituted of approximately 640 mg g^−1^ glucose, 140 mg g^−1^ galactose, 50 mg g^−1^ mannose, 80 mg g^−1^ xylose, 60 mg g^−1^ arabinose, 10 mg g^−1^ fucose and 20 mg g^−1^ rhamnose. Principal component analysis (PCA) indicated that the variation between the monosaccharide composition of cassava from different feed mills, observed in Table 2, is statistically significant. Through data regression, a *n*-dimensional PCA plot (Fig. 1A) was obtained representing all data from the quantification of seven neutral sugars in five cassava samples, in triplicates, according to *n* principal components. The combination of two principal components represented 93.1% of the original data variation and were thereby sufficient to describe most of the information derived from the complete dataset. As indicated in the PCA loading matrix (Supplementary Table S1), all monosaccharides except galactose contributed significantly to Principal Component 1, and mostly rhamnose and galactose contributed to Principal Component 2.

**Table 2:** Content of insoluble neutral non-starch polysaccharide (NSP) sugars in cassava (n = 3).

Insoluble NSP (mg g^−1^ of cassava)	Cassava sample				
	A	B	C	D	E
Rhamnose	0.82	0.66	0.64	0.86	0.93
Fucose	0.33	0.31	0.28	0.36	0.40
Arabinose	2.13	1.82	1.90	3.06	3.72
Xylose	3.03	3.38	3.03	4.66	6.51
Mannose	1.67	1.93	1.42	2.22	3.37
Galactose	5.99	3.01	4.13	5.32	4.15
Glucose	19.71	24.31	18.28	25.52	32.92
Total	33.67	35.43	29.69	42.00	52.00

In the PCA plot, the control treatments (red) of individual cassava samples, represented by different shapes, were separate from each other, while the triplicates were placed together. This indicated that the neutral sugar composition of the cell wall of cassava samples from different feed mills were statistically different.

After incubation of the cassava samples with NSPase at 400 ppm (4 h, 40 °C, pH 5), the total insoluble NSP content of the cassava samples decreased by circa 10%, from about 40.5 mg g^−1^ to about 36.7 mg g^−1^, with a statistically significant solubilization of rhamnose, arabinose, xylose and galactose (Table 3). Despite differences in NSP composition, the enzyme product performed in a similar manner on all cassava samples. The arrow in the PCA plot indicates the direction in which all samples moved throughout the plot, to the left and the bottom, representing a reduction in the amount of monosaccharides present in the insoluble fraction of cassava NSP after the NSPase treatment at 250 ppm (green) or 400 ppm (blue).

**Table 3:** Enzyme effect on the cell wall polysaccharide content in cassava samples.

Enzyme dosage	Neutral monomer sugars* (mg g^−1^)							
	Rha	Fuc	Ara	Xyl	Man	Gal	Glc	Total
0 (control)	0.93 A	0.34 AB	2.87 A	4.18 A	2.23 A	5.72 A	24.26 A	40.52 A
250 ppm	0.76 B	0.35 A	2.44 B	4.13 AB	2.06 B	4.27 B	24.50 A	38.50 B
400 ppm	0.66 C	0.32 B	2.28 C	4.06 B	2.08 B	3.57 C	23.68 A	36.66 C
Least squares fit (R^2^)	0.95	0.77	0.99	0.99	0.98	0.94	0.96	0.98
Effect of sample (P=)	0.02	0.02	0.02	0.02	0.02	0.02	0.02	0.02
Effect of dosage (P=)	0.02	—	0.02	0.02	0.02	0.02	—	0.02

Neutral sugar content of the insoluble NSP fraction of five cassava samples before and after treatment with the commercial NSPase product at 250 or 400 ppm. *Rha = rhamnose, Fuc = fucose, Ara = arabinose, Xyl = xylose, Man = mannose, Gal = galactose, Glc = glucose. ABC: Different capital letters indicate that values within the same column are statistically different (Tukey-Kramer P < 0.05).

When compared to the control treatment, the NSPase product solubilized uronic acids present in cassava, (Fig. 1B). Enzyme dosage response was not evident in all samples. After NSPase treatment, the concentration of uronic acids solubilized varied between 1.24 and 3.73 mg per g of cassava sample. Compared to the control treatment, the lowest solubilization increase was observed for cassava sample A, from about 1.1 to around 2 mg g^−1^ (+76%); and the highest solubilization increase was observed for cassava sample D, from 1.1 to 3.7 mg g^−1^ (+245%).

### Microscopy – cassava cell wall polysaccharide composition and degradation by NSPase

**Figure 2:**
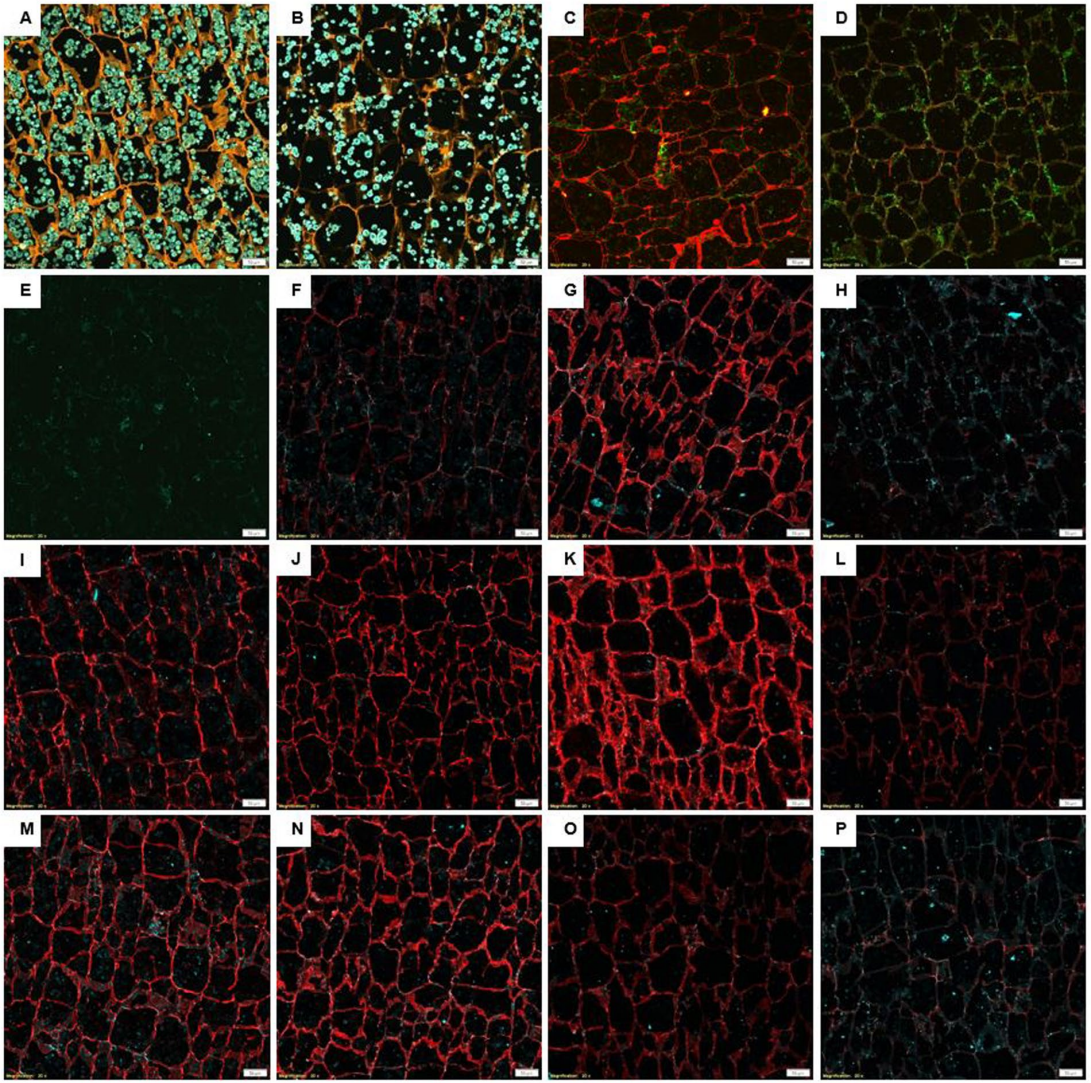
Confocal microscopy pictures of cassava sections. Cassava sections studied under a 20x objective (scale bar = 50 µm). Orange indicates Coriphosphine-O fluorescence. Red indicates Alexa-555 fluorescence. Blue/green indicates auto-fluorescence. (**A)** Control treatment dyed with Coriphosphine-O. (**B)** Treatment with NSPase at 250 ppm and dyed with Coriphosphine-O. (**C)** Control labelled with INRA-RU1. (**D)** Treatment with NSPase at 250 ppm and labelled with INRA-RU1. (**E)** Blank, sample labelled only with the secondary antibody attached to Alexa-555. (**F)** Control labelled with LM19. (**G)** Treatment with NSPase at 250 ppm and labelled with LM19. (**H)** Treatment with NSPase at 800 ppm and labelled with LM19. (**I)** Control treatment labelled with LM25. (**J)** Treatment with NSPase at 250 ppm and labelled with LM25. (**K)** Treatment with NSPase at 400 ppm and labelled with LM25. (**L)** Treatment with NSPase at 800 ppm and labelled with LM25. (**M)** Control treatment labelled with LM5. (**N)** Treatment with NSPase at 250 ppm and labelled with LM5. (**O)** Treatment with NSPase at 400 ppm labelled with LM5. (**P)** β-galactanase 10 ppm treatment labelled with LM5.

A partial cell wall (orange) degradation and a lower density of starch granules (blue/green) was observed after treatment with the NSPase product (Fig. 2A,B).

For the immune-microscopy analysis, a secondary antibody labelled with Alexa-555 was used to detect the presence of primary antibodies bound to specific epitopes on cassava cell walls. No non-specific bindings were present, as indicated by a lack of red signal in the blank sample labelled with the secondary antibody only (Fig. 2E).

There was a notable decrease of Alexa-555 signal (red) in cassava sections after treatment with NSPase at 250 ppm (Fig. 2D), when compared to control treatment (Fig. 2C). This finding indicates that cassava cell wall is rich in rhamnogalacturonan-I (RG-I), and that the enzyme product hydrolyses most of RG-I content into oligosaccharides shorter than six rhamnose-galacturonic acid disaccharide repeats.

The confocal microscopy results indicated that no non-specific bindings were present, as indicated by a lack of red signal in the control sample (Fig. 2E).

The intensity of the Alexa-555 signal on samples labelled with LM20 that targets methyl-esterified homogalacturonan (HG) was weak, and completely disappeared after NSPase treatment at 250 ppm (Supplementary Fig. S1). The intensity of Alexa-555 on sections labelled with LM19 was slightly stronger (Fig. 2F), indicating presence of HG regions that are not esterified in cassava cell wall. When treated with the NSPase at 250 ppm, the LM19 signal increased significantly (Fig. 2G), and when the enzyme dosage was increased to 800 ppm, the signal was almost completely removed (Fig. 2H). The same increase in signal was observed when cassava section was treated with purified pectin methyl esterase.

Figure 2(I) shows a high fluorescence on samples labelled with LM25, indicating presence of xyloglucan in cassava cell walls. No difference was observed in the fluorescence of samples labelled with LM25 after treatment with NSPase dose at 250 ppm (Fig. 2J), and when the enzyme dosage was increased to 400 ppm, an increased fluorescence was observed (Fig. 2K). A further increase of the enzyme dosage to 800 ppm resulted in significant decrease in the fluorescence (Fig. 2L).

Results obtained with LM5, an antibody that targets (1 → 4)-β-D-galactan^16^, were similar to LM25. A high intensity (Fig. 2M) from LM5 was observed on cassava cell walls, the fluorescence intensity did not change after treatment with NSPase at 250 ppm (Fig. 2N). When the enzyme dosage was increased to 400 ppm, the fluorescence signal was considerably reduced (Fig. 2O). To confirm that the reduced antibody signal was due to the degradation of β-galactan, a section of cassava D was treated with a purified β-galactanase at 10 ppm and also labelled with the antibody LM5. This resulted in a close to complete removal of the signal (Fig. 2P).

No signal was detected for linear penta-saccharide of (1–5)-α-L-arabinans and for xylogalacturonan, when using the antibodies LM6 and LM8, respectively (Supplementary Figs S2 and S3). Antibodies were tested on sections of cassava D treated without enzymes and or with NSPase at 250 ppm.

Results indicated the absence of ferulic acid on cassava sections. No signal was detected from any of the antibodies used **(**Supplementary Fig. S4).

### Mass spectrometry – oligosaccharides solubilized by NSPase

It was possible to observe differences regarding the degree of polymerization (DP) and the presence of methyl and acetyl esters between the oligosaccharides profile of different samples. Looking at individual peaks, information about single oligosaccharides was obtained; and the comparison of peak sequences provided an indication of the different activities of the NSPase.

Much higher peak intensity and variety was observed after the enzymatic treatment, when compared to control treatment (Supplementary Fig. S5 - note difference in y-axis scale). Putative assignation with focus on pectin structures of peaks randomly labelled in Supplementary Fig. S5B is provided in Supplementary Table S2.

Treatment with NSPase resulted in hexose oligomers (Δ m/z 162), pentose oligomers (Δ m/z 132), hexuronic acid or ferulic acid oligomers (Δ m/z 176), and heterosaccharides combining hexose, pentose and hexuronic acid units. Mass spectrometry data also indicates that some of the hexuronic acid containing oligosaccharides may be methyl esterified. Methyl esters seem to be removed in pairs (Δ m/z 28) from oligosaccharide chains. Results also indicate that the enzyme product may be able to remove acetyl esters (Δ m/z 42) from hexose oligos and from combining hexose, and hexuronic acid units.

Peak sequences with Δ m/z 176 could represent the removal of either a hexuronic acid or a ferulic acid from the structure. Immuno-microscopy results, where the antibodies LM9 and LM12 were tested against ferulic acid, showed no signal on cassava sections (Supplementary Fig. S4). This indicates that peak sequences with Δ m/z 176 most probably represent removal of hexuronic acid units.

### Cassava treatment with a combination of NSPase and α-amylase

A single sample of cassava (sample D) was chosen to estimate the effect on starch digestion by α-amylase and its possible synergistic effect with the NSPase. The amount of soluble glucose was determined using HPIC. Supernatants of cassava treated without enzymes and with α-amylase at the recommended dosage and the NSPase product at 250 and 400 ppm, and the combination of both were analysed.

**Figure 3:**
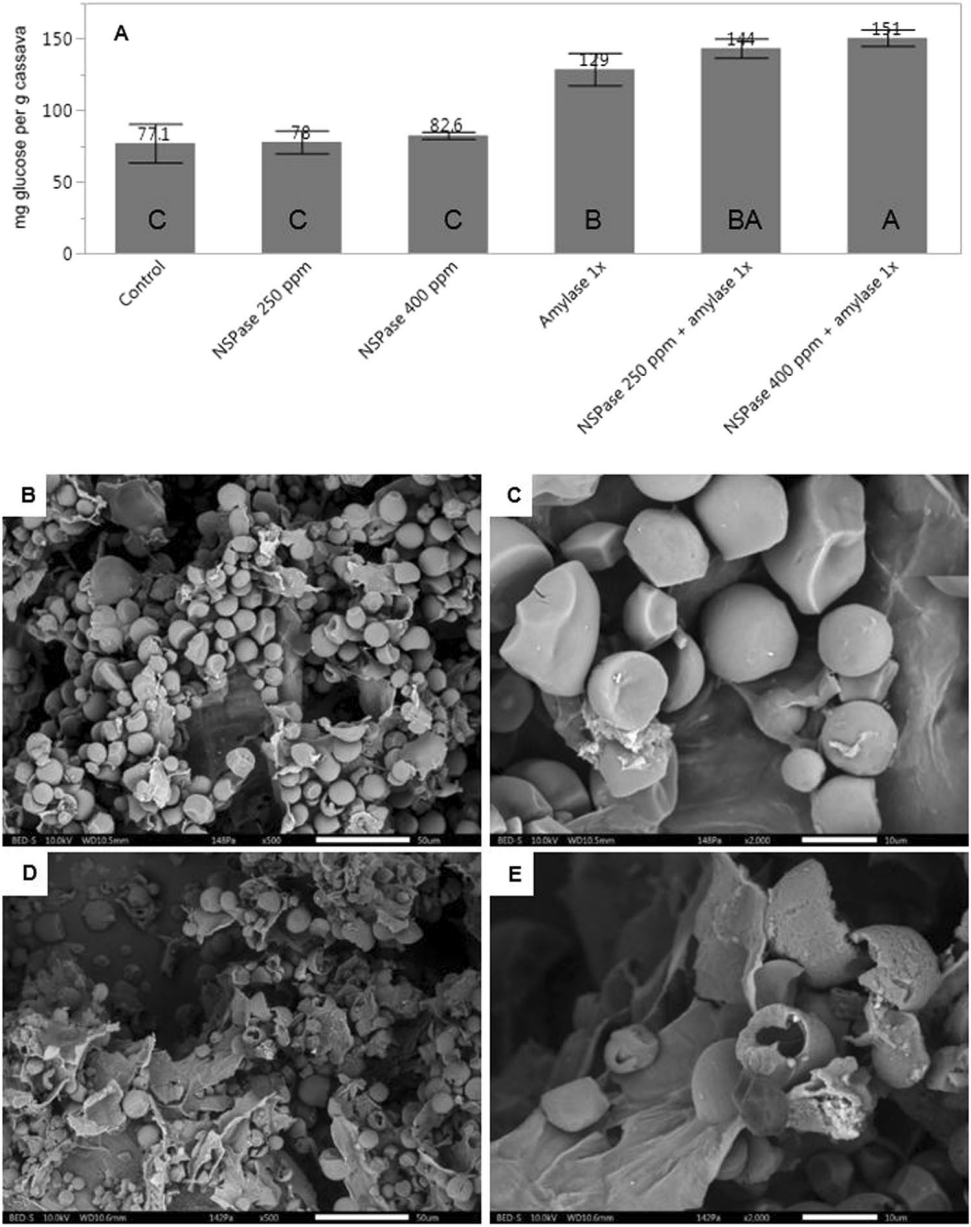
Effect of combinations of enzyme supplementation **(A)** HPIC quantification of glucose in the hydrolysed supernatant, given in mg per g of cassava sample. (**B)** SEM picture of milled cassava D treated without enzymes. Magnification of 500x and scale bar equal to 50 µm. (**C)** SEM picture of milled cassava D treated without α-amylase. Magnification of 2,000x and scale bar equal to 10 µm. **(D)** SEM picture of milled cassava D treated with α-amylase. Magnification of 500x and scale bar equal to 50 µm. (**E)** SEM picture of milled cassava D treated with α-amylase. Magnification of 2,000x and scale bar equal to 10 µm.

After treatment with the α-amylase at a commercial dosage, an increase of almost 70% of soluble glucose was quantified in the supernatant of cassava D compared to control treatment (Fig. 3A).

A numerical, but not statistically significant, increase of solubilized glucose after the treatment with the NSPase enzyme alone was observed (Fig. 3A). An indication of a synergistic effect was seen when both NSPase and α-amylase were combined. There was a statistically significant difference between treatment with α-amylase (129 mg g^−1^, B) alone and combined with NSPase 400 ppm (151 mg g^−1^, A). Around 17% more glucose was solubilized when both enzyme products were applied together, which suggests a higher exposure of starch granules to the activity of α-amylases when approximately 10% of cassava cell walls were enzymatically solubilized.

To investigate the physical properties of cassava starch granules and visualize the effect of the α-amylase, ground samples treated without or with the enzyme were analysed by using a Scanning Electron Microscope (SEM). Cassava D starch granules were about 10 µm in diameter and round, with a smooth surface and sometimes with inward curvatures (Fig. 3B,C). Some of the shape deformation or granule degradation could be an effect of sample preparation, lyophilization, or incubation with buffer at 40 °C for 4 h. When treated with a commercial α-amylase, cracks and big holes on the starch granules surface were observed (Fig. 3D,E).

## Discussion

Compositional analyses of the cassava samples were conducted and results were generally consistent with values reported in the literature for starch and crude protein^9,17–20^, with minor variations between samples, except for cassava E. Fat content values were intermediate to those found in literature^21,22^ while crude fibre content was within the range observed by others^11,23,24^. An elevated content of crude fibre in one of the samples (E) compared to the other samples could have been caused by a higher peel to tuber ratio, since the peel may contain more than four-fold more fibre than the root^24^, and as also indicated by a notably higher ash content in this sample.

In the current study the average content of neutral cell wall sugars for the five samples followed the same concentration order in all samples and was consistent with data reported by others from lowest to highest content of NSP components: fucose, rhamnose, mannose, arabinose, xylose, galactose and glucose^11,23,25^. Compared to literature values, the samples A-E had similar concentrations of rhamnose, a slightly higher arabinose content but a notably higher content of mannose and a markedly lower galactose content. The highest content of total NSP glucose (which included cellulose) was higher than that previously communicated in literature. This could be an artefact caused by resistant starch which may be present in cassava^9,26,27^.

The monosaccharide composition determined by NSP analyses served as a guide for immune-microscopy studies. Paraffin embedding was used since sections can be de-paraffined and thereby enable the access of the enzymes to the substrates. This technique has been reported as useful when investigating the enzymatic solubilization of substrates^28^. Presence of high concentrations of glucose and xylose in the NSP fraction in cassava samples A-E may indicate the presence of xyloglucan polysaccharides, the main hemicellulosic component of dicotyledonous plants^25,29^. This was corroborated by immuno-microscopy, by labelling cassava sections with LM25, that targets specific epitopes in xyloglucan structure^30^. The results confirmed the presence of xyloglucan in cassava cell wall, and the ability of the NSPase to solubilize this polysaccharide.

Galactose, originating from galactan polysaccharides that are found in plant cell walls as sidechains of rhamnogalacturonan-I and arabinogalactan proteins, was the second most abundant neutral NSP sugar in the samples. The presence of galactan in cassava cell wall was demonstrated by the use of the monoclonal antibody LM5, that targets (1 → 4)-β-D-galactan. Subsequently, the antibodies LM9 and LM12, that targets feruloylated (1 → 4)-β-D-galactan and ferulic acid in pectic structures were tested. As expected, no signal was detected from these antibodies, confirming that the polysaccharides of cassava are not feruloylated. Therefore, the Δ m/z 176 sequences observed in the MS spectra most likely represents hexuronic acid. The presence of hexuronic acids was confirmed by the HPIC analyses, and treatment with NSPase resulted in an increased content of soluble uronic acids, that were believed to be galacturonic acids originating from the pectin structure.

The abundance of pectin structures in cassava cell walls was confirmed by colouring with Coriphosphine-O, which binds to acidic polysaccharides, including pectin^31^. The Coriphosphine-O signal was reduced after enzyme treatment, indicating that the NSPases were able to partially hydrolase pectic polymers present in the fibre matrix.

To obtain more information about cassava pectin, the antibodies LM19 and LM20 were used to identify un-esterified and methyl-esterified homogalacturonan regions, respectively. Results obtained with antibodies LM20 indicated that, either the cassava samples had a low content of methyl-esterified HG or the antibody was not optimal for targeting HG, since the fluorescence signal was low in samples without enzyme addition. The preferred substrate for pectin lyase is known to be fully methylated polygalacturonan, but cleavage has also been observed in non-methylated pectin^32^. Therefore, pectin lyase activity might be sub-optimal in cassava, considering the prevalence of non-methyl esterified HG in this raw material.

Immuno-microscopy results show the presence of pectin methyl esterases (PME) in the enzyme product that may de-methylate HG and, consequently, reduce its affinity to the antibody LM20 and increase its affinity to LM19. MS results support this hypothesis, since sequences of peaks that differed from each other in a di-methyl ester were found (Δ m/z 28). To the best of our knowledge, the pattern of removing methyl esters in pairs has not been previously reported, and would have to be further investigated. The degree of methylation and methyl distribution along HG segments vary between different plant tissues and along the plant aging process. Methylation has a high impact on pectin physical behaviour, such as thickening and gelling^33^.

Another reason for the increased signal of LM19 after the treatment with NSPase could be an unmasking effect, meaning that the degradation of other polysaccharides present in the cell wall might increase the accessibility of epitopes in de-methylated HG to the antibody LM19. Enzymatic treatments have previously been utilized to provide information about sets of polysaccharides that may block detection of all structures^34^.

After treatment with a higher NSPase dosage, the signal of LM19 was almost completely removed, indicating the hydrolysis of non-methylated HG, probably by the action of polygalacturonases.

A strong signal of Alexa-555 attached to INRA-RU1 was detected in cassava cell walls, indicating that pectin is one of the most abundant polysaccharides in cassava cell wall. The significant decrease of the Alexa-555 signal on enzyme-treated samples confirms a high activity of rhamnogalacturonases in the NSPase product that may break down RG-I into soluble oligosaccharides, or at least modify the epitope structure, hindering the antibody binding.

Arabinans and xylogalacturonans (XGA) are other known pectin structures expected to be found in cassava cell walls^35^, and they can be labelled with antibody LM6 and LM8, respectively. No signal was detected for any of these antibodies. The manufacturer specifies that LM8 does not bind to all xylogalacturonans^36^, therefore it is not possible to assume the absence of XGA in cassava cell wall. The same reason might explain the absence of signal with LM6, since antibodies usually have highly specific epitopes, and complex polysaccharides like pectin vary in their molecular structure between plant species. Additionally, critical interpretation of data is necessary when using commercial antibodies produced to target a polysaccharide in a different plant species^28^.

The analysis of oligosaccharides generated after the treatment of cassava cell wall with a multicomponent NSPase through MS was very complex. Therefore, interpretations were mostly focused in the differences between peak sequences to obtain insights about enzyme activities on cassava cell wall, rather than the numerous possibilities of monosaccharides composition of each peak. Despite data analysis limitations, the much higher amount and diversity of pectin oligosaccharides obtained has an important potential as prebiotics. A putative assignation for some of the identified peaks selected is shown in Supplementary Table S2. A focus on Rhamnogalacturonan-II and Homogalacturonan structures was given due to the major NSP in cassava being pectin. Oligosaccharides from cassava have been previously shown to have a prebiotic effect^37^.

The original morphology of cassava starch granules observed with SEM was in good agreement with what is reported in the literature^38–41^. The effect of an exogenous α-amylase on cassava starch granules was also observed by using a SEM. Results observed with SEM are in good accordance with *in vitro* data. After treatment with the α-amylase product, cracks and big holes in starch granules surface were observed. The degradation of cassava starch granules in the present work was harsher than previously reported in the literature, where mild corrosion of granules surface was observed after treatment with α-amylase for 36 h at 30 °C and pH 6^42^.

Confocal microscopy imaging of samples stained with Coriphosphine-O showed that the cell wall of cassava treated with NSPase was partially degraded and that the samples had a lower density of starch granules when compared to control treatment. Despite differences in composition between cassava samples from different feed mills, around 10% of the total NSP content was solubilized for all samples treated with NSPase at 400 ppm. HPIC analysis showed that the combination of an NSPase and an exogenous α-amylase resulted in an increase of 17% in the content of glucose solubilized from starch, when compared to cassava treated with the α-amylase only. These numbers, that appear to be modest, may still justify the enzyme supplementation of cassava in animal feed. *In vivo* trials should be performed to confirm the improvement in animal performance from an envisaged improved energy availability caused by the release of cell wall entrapped starch granules.

Results show that treatment with a NSPase can partially solubilize cassava cell walls and increase starch release. As a result of the enzyme reactions, oligomers containing mainly uronic acids, galactose, arabinose and rhamnose are released. Oligomers from cassava hemicellulosic structure were previously reported to have prebiotic effect^37^. The results above will hopefully contribute to the investigation of the potential prebiotic effect of oligomers from cassava pectin structure, as well as the exploration of both cassava and NSPases not only in the feed industry, promoting animal health and performance, but also across industries.

## Methods

### Materials

#### Plant materials and samples preparation

Granules of cassava roots from five different feed mills in South East Asia, each of approximately 200 grams, were procured from DSM Nutritional Products, APAC. Samples were identified with a letter (A–E). For wet chemistry assays, samples were milled using a 0.5 mm opening sieve (Ultra Centrifugal Mill ZM 200, Retsch, Germany).

For microscopy, granules were diced into cubes with 0.5 cm edges, fixed in formalin (BiopSafe, Axlab, Denmark), washed with PBS buffer (pH 7.4), dehydrated with sequential ethanol solutions with increased concentrations (from 50 to 99.9%), and submerged in HistoChoice Clearing Agent. Subsequently, samples were embedded in paraffin (Paraplast, Sigma-Aldrich, USA) and sectioned in 5 µm slices (Microm HM 355S, Thermo Fisher Scientific, USA). De-paraffination was done in HistoChoice, followed by a decreased graded ethanol solution series, from 99.9 to 0% in water.

#### Enzymes

RONOZYME VP (DSM Nutritional Products, Switzerland) is a multicomponent cell wall degrading carbohydrase (NSPase), produced by the fermentation of *Aspergillus aculeatus*. It is known to have various hemicellulases and pectinases activity (Supplementary Table S3). RONOZYME HiStarch (DSM Nutritional Products, Switzerland) is a monocomponent α-amylase, produced by the fermentation of *Bacillus amyloliquefaciens*. Amyloglucosidase (AMG) was obtained from Megazyme (Ireland). Experimental β-galactanase was obtained from Novozymes A/S (Denmark).

#### Chemicals

Formalin (BiopSafe) was purchased from Axlab (Denmark), and Potassium hydroxide 7.5 M was from Ampliqon (Denmark). All other chemicals were from Sigma-Aldrich (USA).

#### Antibodies

All monoclonal antibodies were purchased from Plant Probes (UK), except INRA-RU1, that originated from INRA (France).

### Analysis

#### Compositional analysis

Gross chemical analysis was conducted by Eurofins Scientific (Belgium). Moisture, crude protein, crude fat, crude ash and crude fibre contents were determined according to official reference methods^43^. Enzymatic starch was determined following the Nordic Feed Evaluation System reference method^44^. The Kjeldahl conversion factor used to estimate crude proteins was 6.25.

#### NSP analyses for solubilization of cassava cell wall

Triplicate samples were incubated at 40 °C for 4 hours in a 0.1 M NaAc buffer containing 5 mM calcium and pH equal to 5.0 ± 0.05, both in the absence and presence of the cell wall degrading product at 250 and 400 ppm. To separate the insoluble fraction of NSP, samples were centrifuged at 1,400 g at 5 °C for 15 minutes and the supernatant was discarded. The pellets were re-suspended twice in 0.1 M NaAc buffer and supernatants were discarded after centrifugation. Subsequently, NSP analyses were performed according to The Uppsala Method^45^. The neutral sugars that were derivatized to alditol acetates were quantified by gas chromatography (Agilent, USA). The software used to integrate the chromatograms peaks was ChemStation (Agilent, USA).

#### Mass spectrometry

Milled cassava sample was incubated at 40 °C for 4 hours in a 0.1 M NaAc buffer containing 5 mM calcium and pH equal to 5.0 ± 0.05, both in the absence and presence of the NSPase product at 250 ppm. Afterwards, samples were centrifuged, and the supernatant was filtered (1.2 µm) and freeze-dried (Heto PowerDry LL3000, Thermo Fisher Scientific, USA). Subsequently, 10 mg of lyophilizate was resuspended in 1 ml of Milli-Q water. Aliquots of 0.7 µl of sample mixed 1:1 with THAP matrix (20 mg/ml in a 50% methanol solution) was applied to an anchorchip target plate. The analysis was run on MALDI MS (UltrafleXtreme TOF/TOF, Bruker, Germany), equipped with a 355 nm Smartbeam II Nd:YAG (neodymium-doped yttrium aluminium garnet) laser operated at 2 kHz in reflectron positive-ion mode (reflector 5.7x). Mass range was set between 100–4500 m/z. Maltodextrin (1 mg/ml) was used for mass calibration.

Data acquisition and analysis of oligosaccharides generated by treatment of cassava with the NSPase were performed using the Flex software suite (FlexControl 3.4 and FlexAnalysis 3.4, Bruker Daltonik, Germany).

### Microscopy

#### Enzyme treatment

De-paraffined cassava sections on the glass slides were covered with 100 µl of enzyme solution and incubated for 3 hours at 40 °C on a heating block W10 (VWR, USA). The final enzyme concentration was calculated in ppm of enzyme product per amount of cassava sample. Enzyme solutions were prepared in 0.1 M sodium-acetate buffer with 5 mM calcium at pH 5.0. A control treatment was also included, where cassava section was incubated with 100 µl of buffer (without enzyme) under the same condition. After incubation, the slides were washed in Milli-Q water and dried.

#### Staining

De-paraffined cassava sections were stained with 0.1% (water) coriphosphine-O for 10 min. Excess dye was washed out with Milli-Q water. Coriphosphine-O is known to stain pectin^31^.

#### Immunolabeling

Immunochemistry was performed on treated cassava sections by blocking samples with 5% skimmed milk in 0.01 M PBS buffer (pH 7.4) and washed with PBS buffer. Subsequently, sections were incubated with monoclonal antibodies (Table 4) diluted 10 times in 5% skimmed milk in PBS buffer. Afterwards, samples were washed with PBS buffer and incubated with a 300-fold diluted anti-rat IgG linked to Alexa-555 fluorophore in PBS buffer with 5% skimmed milk and 1% BSA. Cover-glass was placed on top of labelled section after adding a drop of anti-fading agent Citifluor AF1 (Agar Scientific, UK)^16^. Negative controls were prepared by incubating cassava sections with the secondary antibody after blocking with 5% milk.

**Table 4:** Monoclonal antibodies tested against cassava cell wall, their corresponding epitopes and source animal.

Antibody	Epitope	Origin	Reference
LM5	(1 → 4)-β-D-galactan	Rat IgG	PlantProbes^16^
LM6	(1 → 5)-α-L-arabinan	Rat IgG	PlantProbes^46^
LM8	Xylogalacturonan - recognizes a specific epitope of a xylogalacturonan pectic polysaccharide, does not bind to all xylogalacturonans	Rat IgM	PlantProbes^36^
LM9	Feruloylated (1 → 4)-β-D-galactan	Rat IgM	PlantProbes^47^
LM12	Feruloylated polymers - recognizes an epitope containing ferulic acid that is a structural feature of the pectic polymers	Rat IgG2c	PlantProbes^30^
LM19	Homogalacturonan - binds strongly to un-esterified homogalacturonan	Rat IgM	PlantProbes^48^
LM20	Homogalacturonan - requires methyl-esters for recognition of homogalacturonan and does not bind to un-esterified homogalacturonan	Rat IgM	PlantProbes^48^
LM25	Xyloglucan - binds to the XLLG, XXLG and XXXG oligosaccharides of xyloglucan	Rat IgGM	PlantProbes^30^
INRA-RU1	Backbone of Rhamnogalacturonan I - recognizes unbranched regions of RG-I and requires at least 6 disaccharide backbone repeats for binding	Mouse IgM	INRA^49^

#### Confocal microscopy

Treated cassava sections stained with coriphosphine-O were analysed under the IX83 Confocal Laser Scanning Microscope (Olympus, Japan). Samples were excited at 488 and 561 nm and emission was detected in 490–530 and 600–650 nm spectra, respectively. All pictures were taken with a magnification of 20x.

Confocal images of immunolabelled cassava sections treated with buffer (control) and with the NSPase product (dosed at 250 and 400 ppm) were taken using a IX83 Confocal Laser Scanning Microscope (Olympus, Japan). All samples were analysed with a 20x objective. Alexa-555 fluorescein-signal was excited at 561 nm (green) and detected in an emission spectrum between 570–620 nm, designated as red in the software. Cassava samples were excited at 405 nm (blue) and autofluorescence emission was observed within 429–470 nm, designated as green in the software.

The images were processed using the FV31S-SW software (Olympus, Japan).

#### Scanning electron microscopy

After incubation of milled cassava with the α-amylase product at 10x commercial dosage, samples were centrifuged at 1,400 g and 5 °C for 15 min (Multifuge X3R, Thermo Fisher Scientific, USA), and pellets were lyophilized (Heto PowerDry LL3000, Thermo Fisher Scientific, USA). Subsequently, samples were fasted on 12.5 × 10.0 mm sized cylinder stubs (Agar Scientific, UK) with carbon-based double-sided tape Leit-tabs (Agar Scientific, UK). Loose particles were removed with vacuum before coating the samples with gold (Q150R Rotary-pumped coater, Quorum Technologies Ltd, UK). Visualization of samples surface was done using a JSM IT300 scanning electron microscope (JEOL Ltd., Japan), equipped with a tungsten filament and a BED-S (secondary backscattered electron) detector. The accelerating voltage was set to 10.0 kV and vacuum was between −142 and −148 Pa. Magnification of 500x and 2,000x were applied.

#### Solubilized glucose determination by high performance anion exchange chromatography combined with pulsed amperometric detection (HPAEC-PAD)

Cassava D was incubated at 40 °C for 4 hours in a 0.1 M NaAc buffer containing 5 mM calcium and pH equal to 5.0 ± 0.05, without enzymes, with the NSPase product at 250 and 400 ppm, with the $a$-amylase at the recommended dosage, and with the combination of both products (n = 3).

After centrifugation at 1,400 g and 5 °C for 15 min (Multifuge X3R, Thermo Fisher Scientific, USA), supernatants were hydrolysed with 1.3 M HCl for 1 hour at 99 °C in water bath (SC150, Thermo Fisher Scientific). Subsequently, hydrolysates were neutralized with 1.3 M NaOH, filtered through a 0.20 μm pore size Millex Syringe Nylon Filter (Merck Millipore, USA) and diluted in Milli-Q water. HPAEC-PAD was conducted using a ICS-5000+ Dionex equipped with a guard column CarboPac PA210G and an analytical column CarboPac PA210 (Thermo Fisher Schientific, USA). Column temperature was set to 40 °C, compartment to 30 °C, and sampler to 10 °C. The flow rate was isocratic (0.200 ml min^−1^), and eluent concentration (1 mM KOH) was constant during the first 8 min of each run. From minute 8 to 13, eluent concentration was kept at 100 mM. And from minute 13 to 28, it was set back to 1 mM. The sample injection volume was 0.4 µl. Electrochemical Detector was used with AgCl as reference electrode. Data acquisition and peaks integration were done using a Chromaleon 7. To quantify the monosaccharides present in the samples, peak areas were interpolated from a standard curve.

#### Uronic acids determination with HPAEC-PAD

Cassava D was incubated in triplicates at 40 °C for 4 hours in a 0.1 M NaAc buffer containing 5 mM calcium and pH equal to 5.0 ± 0.05, without enzymes or with the NSPase product at 250 and 400 ppm. Afterwards, samples were centrifuged, stored, hydrolysed and filtered as described in the item above.

HPAEC-PAD analyses were performed using the same column, temperatures and flow rate as described in the item above. The eluent concentration (0.1 M NaAc buffer) was constant during the first 5 min of each run. From minute 5 to 5.5 the eluent concentration was increased to 0.3 M, and kept constant until minute 11. From minute 11 to 11.5, the NaAc buffer concentration was decreased to 0.1 M, and kept constant until minute 20. The sample injection volume was 0.4 µl. Electrochemical Detector was used with AgCl as reference electrode. Data acquisition and peaks integration were done using a Chromaleon 7. Galacturonic and glucuronic acids were quantified together as uronic acids, since they eluted from the column together.

#### Data analysis and statistics

Data processing was done utilizing Excel 2016 (Microsoft, USA) and JMP 13.2.0 (SAS Institute Inc., USA). Statistically significant differences were determined by the Tukey-Kramer test at α = 0.05, as provided in the ANOVA procedure in the statistical package.

## Supplementary information

Supplementary materials

## Data Availability

The datasets generated and analysed during the current study are available from the corresponding author on reasonable request.
