# Supplementary material for: Cassava cell wall characterization and degradation by a multicomponent NSP-targeting enzyme (NSPase)
Source: Sci Rep. 2019 Jul 12;9:10150. doi: 10.1038/s41598-019-46341-2 (PMC6626134; doi:10.1038/s41598-019-46341-2)
Supplement: Supplementary file 1 — Supplementary materials [file 41598_2019_46341_MOESM1_ESM.docx]

Cassava cell wall characterization and degradation by a multicomponent NSP-targeting enzyme (NSPase)

Larissa Staack^1,2^, Eduardo Antonio Della Pia^1^, Bodil Jørgensen^2^, Dan Pettersson^1^, Ninfa Rangel Pedersen^1,^*

^1^ Novozymes A/S, Krogshoejvej 36, 2880 Bagsværd, Denmark

^2^ University of Copenhagen, Department of Plant and Environmental Sciences, Thorvaldsensvej 40, 1871 Frederiksberg, Denmark

* [nrp@novozymes.com](mailto:nrp@novozymes.com)

Supplementary material

Tables

***Table S1:* PCA loading matrix.**

| **Neutral sugar** | **Prin1** | **Prin2** |
| --- | --- | --- |
| Rhamnose | 0,76622 | 0,59502 |
| Fucose | 0,87567 | -0,00320 |
| Arabinose | 0,96572 | 0,13442 |
| Xylose | 0,95531 | -0,24095 |
| Mannose | 0,94858 | -0,24226 |
| Galactose | 0,20779 | 0,96646 |
| Glucose | 0,90588 | -0,35739 |

***Table S2:* MS pectin oligosaccharides.** Putative assignation with focus on pectin structures of selected peaks labelled in Supplementary Fig. S5, B. In the table, G = galacturonic acid residue, R = rhamnose residue, RG-II = rhamnogalacturonan-II, HG = homogalacturonan, Me = methyl ester residue, Ac = acetyl ester residue.

| **Pectin** | | | |
| --- | --- | --- | --- |
| **Mw** | **NSP** | **Backbone** | **Decoration** |
| **569,263** | **RG-II** | GRG | Me Na^+^ |
| **569,263** | **HG** | GGG | Na^+^ |
| **723,583** | **RG-II** | RGRG | 2 Me H^+^ |
| **723,583** | **HG** | GGGG | H^+^ |
| **751,619** | **RG-II** | RGRG | Me Ac H^+^ |
| **751,619** | **HG** | GGGG | 2Me H^+^ |
| **899,596** | **RG-II** | GRGRG | 2 Me H^+^ |
| **899,596** | **RG-II** | RGRGR | Ac H^+^ |
| **899,596** | **HG** | GGGGG | H^+^ |
| **1075,659** | **RG-II** | RGRGRG | 3 Me H^+^ |
| **1075,659** | **RG-II** | RGRGRG | Ac H^+^ |
| **1075,659** | **HG** | GGGGGG | H^+^ |

***Table S3:* Molecular composition of RONOZYME**^®^ **VP.** List of enzymes identified in four independently produced batches of RONOZYME^®^ VP determined by LC/MS^51^. Relative protein concentrations were calculated from the Mascot EMPAI values^51^.

| **Enzyme activity** | **Relative abundance** |
| --- | --- |
| Cellulose β-1,4-endoglucanase/β-1,3(4)-endoglucanase | High |
| Cellobiohydrolase | Medium |
| β-1,4-glucosidase | Medium |
| Xyloglucan β-1,4-endoglucanase | Medium |
| α-arabinofuranosidase | Medium |
| α-xylosidase | Medium |
| α-fucosidase | Low |
| α-1,4-galactosidase | Medium |
| β-1,4-galactosidase | Medium |
| β-1,4-endoxylanase | Low |
| β-1,4-xylosidase | Medium |
| α-arabinofuranosidase | Medium |
| α-1,4-galactosidase | Medium |
| β-1,4-galactosidase | Medium |
| β-1,4-endomannanase | Medium |
| β-1,4-galactosidase | Medium |
| α-1,4-galactosidase | Medium |
| Rhamno/polygalacturonases | High |
| α-rhamnosidase | Low |
| α-arabinofuranosidase | Medium |
| Exoarabinanase | Low |
| β-1,4-endogalactanase | Medium |
| β-1,4-galactosidase | Medium |
| Pectin lyase | Medium |
| Rhamnogalacturonan lyase | Low |
| Pectin methyl esterase | High |
| Rhamnogalacturonan acetyl esterase | Low |

Figures

**A**

**B**


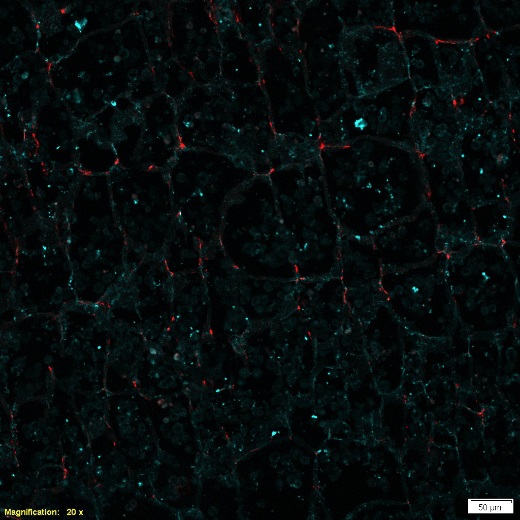

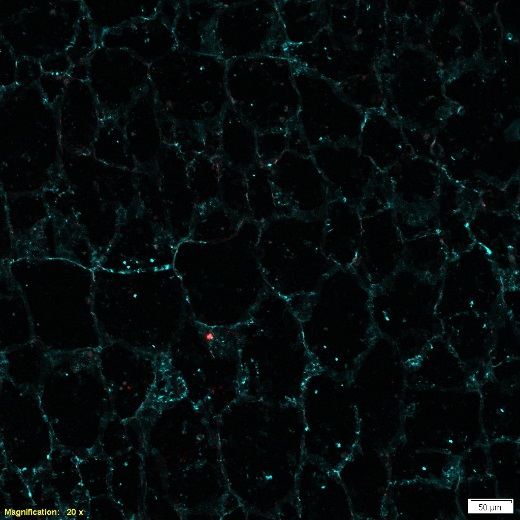


***Fig. S1:*** Slices of cassava D labeled with LM20 after treatment **A)** without enzymes and **B)** with NSPase at 250 ppm. Magnification 20x, scale bar equal to 50 µm.

**B**

**A**


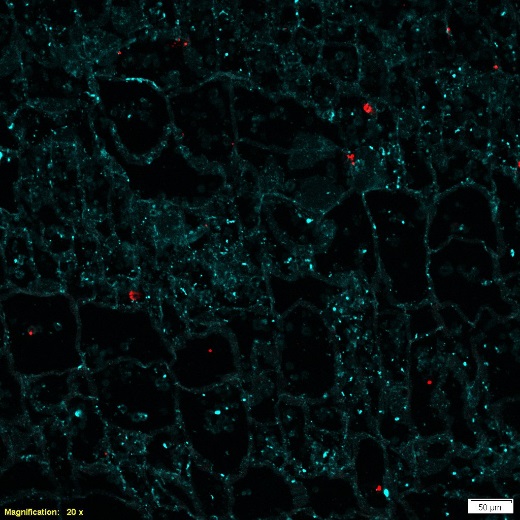

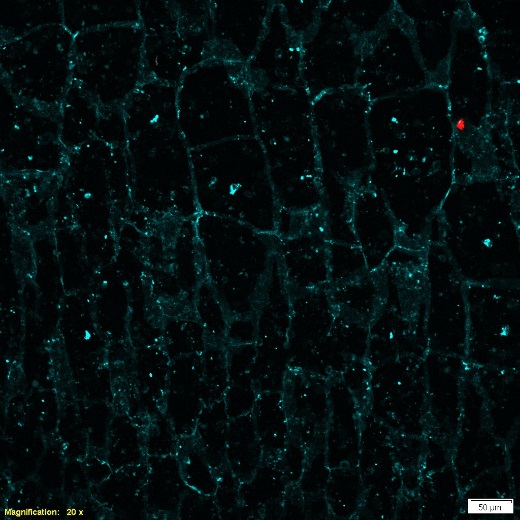


***Fig. S2:*** Slices of cassava D labeled with LM6 after treatment **A)** without enzymes and **B)** with NSPase at 250 ppm. Magnification 20x, scale bar equal to 50 µm.

**B**

**A**


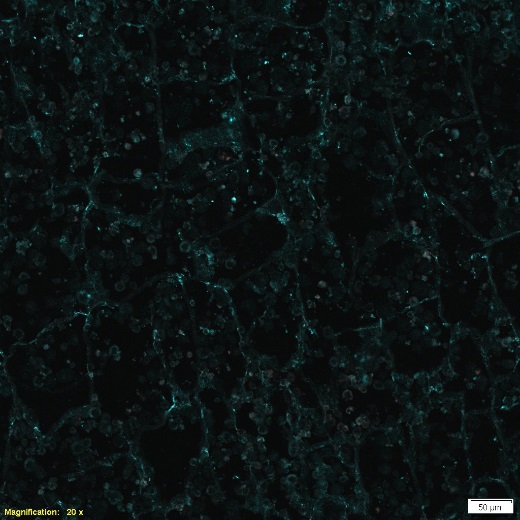

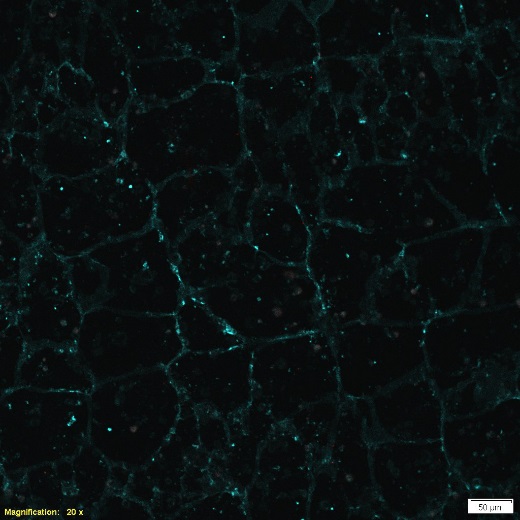


***Fig. S3:*** Slices of cassava D labeled with LM8 after treatment **A)** without enzymes and **B)** with NSPase at 250 ppm. Magnification 20x, scale bar equal to 50 µm.

**A**

**B**


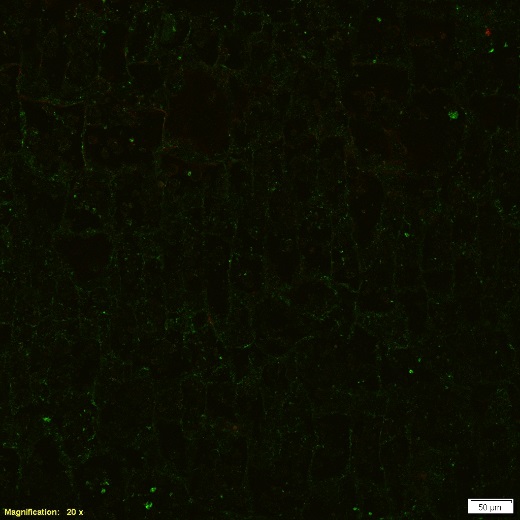

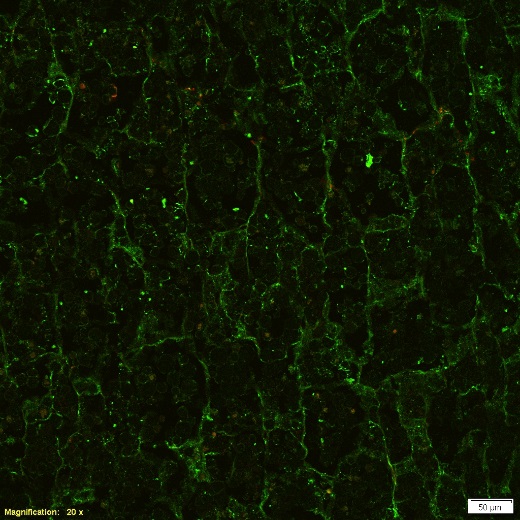


***Fig. S4:*** Slices of cassava D treated without enzymes labeled with **A)** LM9 and **B)** LM12. Magnification 20x, scale bar equal to 50 µm.

**A**

**B**

***Fig. S5:*** Mass spectrum of cassava D supernatant after treatment **A)** without enzymes, and **B)** with NSPase at 250 ppm. Spectra were zoomed in from 480 to 1300 m/z area.
